# The double benefit of *Spalax* p53: surviving underground hypoxia while defying lung cancer *cells in vitro* via autophagy and caspase-dependent cell death

**DOI:** 10.18632/oncotarget.11443

**Published:** 2016-08-20

**Authors:** Martin Ellis, Orly Stern, Osnat Ashur-Fabian

**Affiliations:** ^1^ Translational Hemato-Oncology Laboratory, Hematology Institute and Blood Bank, Meir Medical Center, Kfar-Saba, 4428164, Israel; ^2^ Sackler Faculty of Medicine, Tel Aviv University, Tel Aviv, 69978, Israel; ^3^ The Department of Human Molecular Genetics and Biochemistry, Sackler Faculty of Medicine, Tel Aviv University, Tel Aviv, 69978, Israel

**Keywords:** spalax, p53, lung cancer, autophagy, caspases

## Abstract

The blind subterranean mole rat, *Spalax ehrenbergi*, is a model organism for hypoxia tolerance. This superspecies have adapted to severe environment by altering an array of hypoxia-mediated genes, among which an alteration in the *p53* DNA binding domain (corresponding to R174K in humans) that hinders its transcriptional activity towards apoptotic genes. It is well accepted that apoptosis is not the only form of programmed cell death and that mechanisms that depend on autophagy are also involved. In the current work we have extended our research and investigated the possibility that *Spalax* p53 can activate autophagy. Using two complementary assays, we have established that over-expression of the *Spalax* p53 in p53-null cells (human lung cancer cells, H1299), potently induces autophagy. As *Spalax* is considered highly resistant to cancer, we further studied the relative contribution of autophagy on the outcome of H1299 cells, following transfection with *Spalax* p53. Results indicate that *Spalax* p53 acts as a tumor suppressor in lung cancer cells, inducing cell death that involves autophagy and caspases and inhibiting cell number, which is exclusively caspase-dependent. To conclude, the *Spalax* p53 protein was evolutionary adapted to survive severe underground hypoxia while retaining the ability to defy lung cancer.

## INTRODUCTION

The blind subterranean mole rat, *Spalax ehrenbergi* superspecies, is a model organism for hypoxia tolerance. *Spalax*, has an exceptionally long life-span, spent entirely in underground sealed burrows under extreme hypoxic conditions [1, 2]. It survives 6% O_2_ and 7% CO_2_ in nature, and 3% O_2_ and up to 15% CO_2_ for approximately 11 h in laboratory settings, whereas Rattus die after 2–4 h in similar conditions [3].

*p53* is a master gene orchestrating an array of tumor suppressing activities in response to a variety of stress conditions [4, 5], including hypoxia. p53 is among the most mutated proteins in human cancers. The *Spalax p53* binding domain was shown to contain a specific amino acid substitution (corresponding to R174K in humans) with a bias against the transcription of apoptotic genes while favoring cell arrest and DNA repair genes [6]. This mechanism is believed to contribute to *Spalax*'s hypoxia adaptation by escaping from apoptosis [6–9].

It is now well accepted that apoptosis is not the only form of programmed cell death and that mechanisms that depend on autophagy are also implicated [10, 11]. Thus, we were specifically interested to explore the ability of the *Spalax* p53 to induce autophagy. Using two complementary autophagy assays, we have established that *Spalax* p53 is able to potently activate autophagy in the p53-null human lung cancer cells (H1299). As the blind mole rat is highly cancer resistant [12], we were further interested to explore the whether the mechanisms that *Spalax* have evolved in the *p53* gene to survive hypoxia, might have an advantage relating to cancer resistance. Our results established that *Spalax* p53 acts as a tumor suppressor, inhibiting H1299 cell number that is exclusively caspase-dependent, while inducing cell death that involves both autophagy and caspases. To the best of our knowledge this is the first demonstration of such an activity by the *Spalax* p53 protein, which was evolutionary adapted to survive severe underground hypoxia while retaining the ability to defy cancer.

## RESULTS

### Spalax p53 activates autophagy in lung cancer cells

We have previously shown that *Spalax* p53 evolves a substitution in the DNA binding domain that hinders its transcriptional activity towards apoptotic genes [6, 7]. In the current work we have extended our research and investigated the possibility that *Spalax* p53, retained the ability of the human p53 [13] to activate autophagy.

**Figure 1:**
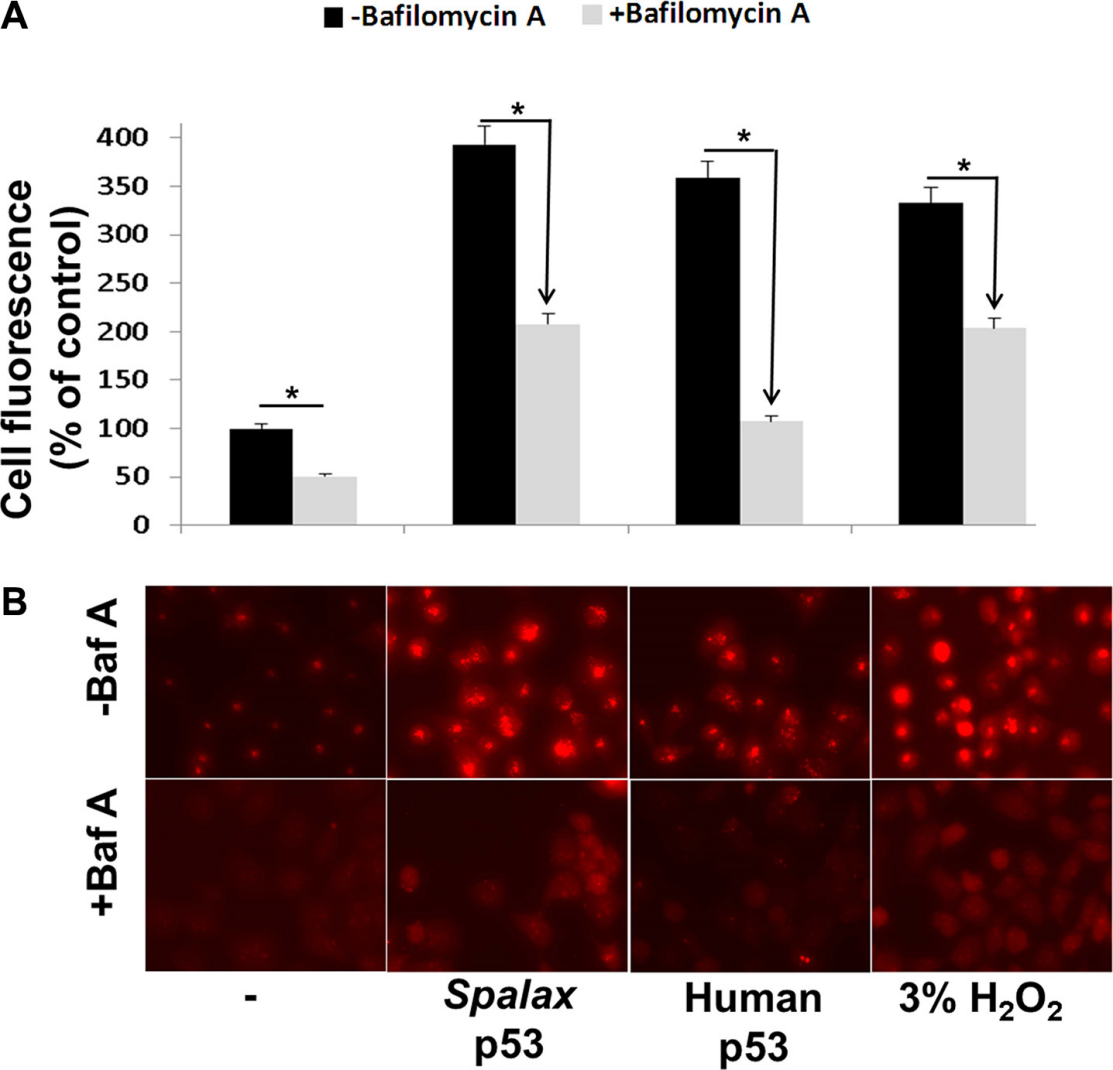
Human and *Spalax* p53 initiate authopagy in lung cancer cells H1299 cells were transfected with the human or *Spalax* p53 plasmids for 72 hours, after which the cells were stained with acridine orange. Fluorescence in four fields per well were counted by fluorescence microscope equipped with a camera and an average value was calculated using NIH ImageJ software. 3% hydrogen peroxide (H_2_O_2_) was used as positive control. The experiments were conducted in the presence and absence of an autophagy inhibitor, Bafilomycin A1 (Baf A). (**A**) Cell fluorescence (% of control) for the different treatments. (**B**) Representative fluorescent microscopy images. Experiments were repeated twice. Results are presented as % of empty vectors (pCMV for the human wild type p53; pCDNA3 for the *Spalax* p53. *p < 0.05.

The extent of autophagy was studied in the p53-null human non small cell lung cancer model (H1299), a valuable platform for researching p53-related activities. *Spalax* p53 plasmids were transiently transfected into the cells. Human wild type p53 plasmid was used for comparison. The cells were stained, 72 hours post transfection, with the lysosomotropic agent, acridine orange. This dye accumulates in acidic compartment and is used to detect and quantify acidic vesicular organelles (AVO), characteristic of autophagy activation. This accumulation is observed as a bright red fluorescence, which is proportional to the degree of authophagy in the cells. For normalization, transfection with appropriate empty vectors (pCMV for the human p53 or pCDNA3 for the *Spalax* p53), were used (Supplementary Figure S1). Fluorescence was recorded by a fluorescence microscope equipped with a camera and quantified using NIH ImageJ software. Results (Figure 1A) have indicated a low level of basal authophagy in the non-transfected cells, which was induced by 3.9-fold following transfections with the *Spalax* p53 plasmid. The human p53 induced 3.6-fold increase in autophagy, while a 3.3-fold was observed by the positive control, 3% hydrogen peroxide (H_2_O_2_). The experiments were further conducted in the presence of an autophagy inhibitor, Bafilomycin A1, which effectively reversed autophagy induction in all cases, indicating specificity. Representative fluorescent microscopy images are presented in Figure 1B.

**Figure 2:**
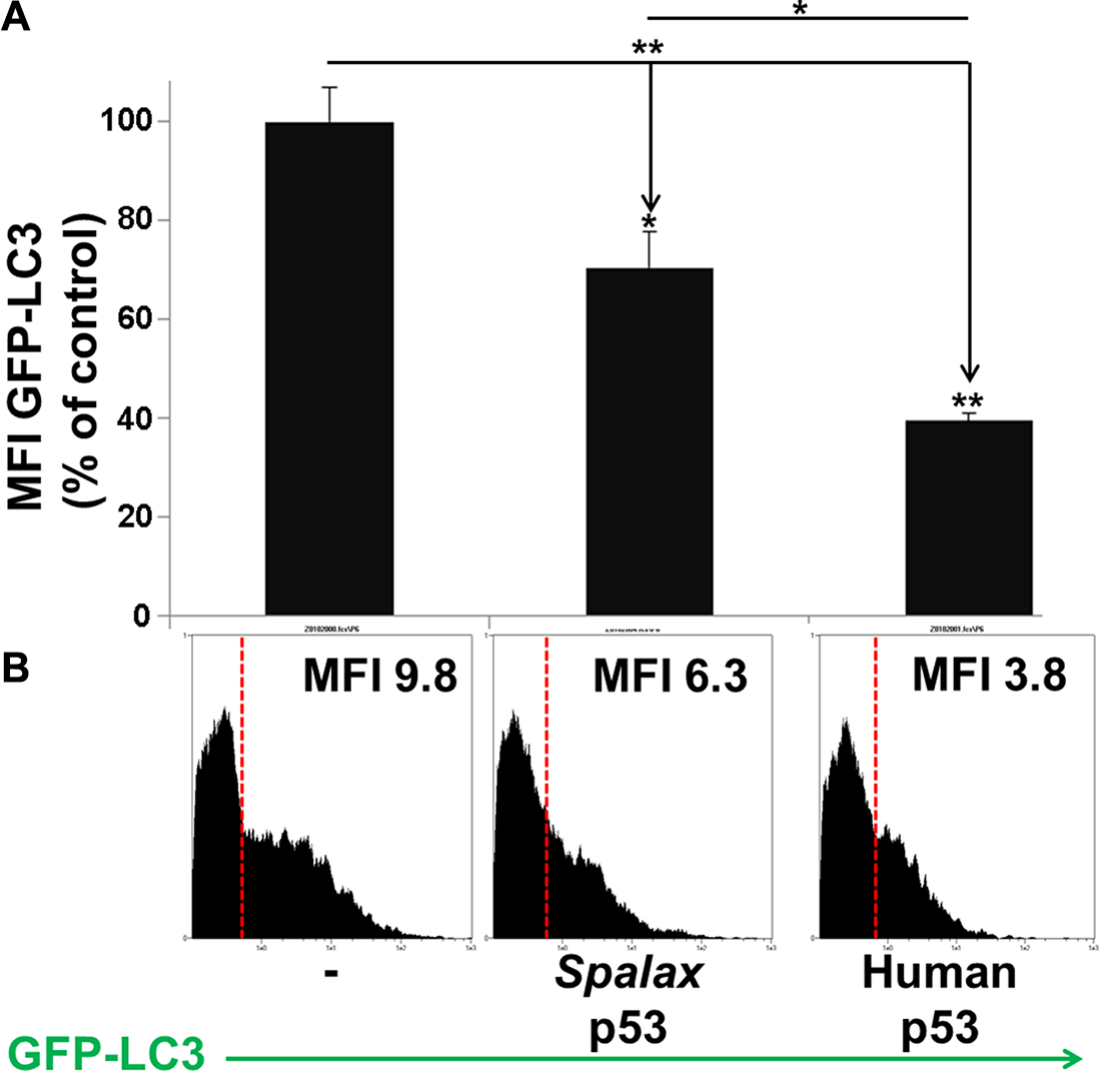
H1299 cells were transfected with the human or *Spalax* p53 plasmids in the presence or absence of GFP-LC3 As controls, cells tranfected with GFP-LC3 alone or co-transfected with empty vectors (pCMV or pCDNA3), were used. Changes in total intracellular GFP-LC3 fluorescence intensity were measured 72 hours post transfection by flow cytometry. (**A**) Mean Fluorescence Intensity (MFI) for each treatment is presented. (**B**) Representative FACS histogram with MFI values for each treatment is depicted. Results were repeated twice, in duplicates. Results are presented as % of empty vectors (pCMV for the human wild type p53; pCDNA3 for the *Spalax* p53). *p < 0.05, **p < 0.005.

Next, the effect of *Spalax* p53 on authophagy in the cells was further evaluated by measuring the turnover of intra-cellular GFP-LC3. The change in GFP levels reflects an autophagic flux and is used as an indicator of cellular autophagic activity in living cells. Increased autophagic flux is expected to result in a progressive delivery of GFP-LC3 to autolysosome, where this substrate undergoes degradation. Therefore, enhanced autophagic flux is detected as a decrease in total cellular GFP signal. H1299 cells were transfected with *Spalax* p53 plasmid in the presence or absence of GFP-LC3. Human wild-type p53 was used for comparison. As controls, cells tranfected with GFP-LC3 alone or co-transfected with appropriate empty vectors (pCMV or pCDNA3), were used (Supplementary Figure S2). Changes in total intracellular GFP-LC3 fluorescence intensity were measured 72 hours post transfection by a MACSQuant flow cytometer. Mean Fluorescence Intensity (MFI) for each treatment was determined and is presented in Figure 2A. Compared to transfection with the empty vectors, a 30% decrease in GFP signal intensity was observed in H1299 cells transfected with the *Spalax* p53. Using this method, transfection with the human wild-type p53 resulted in a 60% decrease in GFP intensity. The difference between the *Spalax* and human p53′s were significant (*p* < 0.05). Representative flow cytometer histograms are presented for each treatment (Figure 2B). Taken together, we have demonstrated, by two complementary methods, albeit with different potencies in comparison to the human p53, that *Spalax* p53 is able to induce authophagy in human lung cancer cells.

### Spalax p53 induces lung cancer cell death that involves authophagy and caspases

The novel observation that *Spalax* p53 is able to induce autophagy in lung cancer cells, led us to explore the involvement of autophagy in an array of cellular activities. H1299 cells were transfected for 72 hours with expression plasmids for *Spalax* p53. Human wild type or mutated p53 (H179R), served for comparison. Transfections with empty vectors, pCMV for human and mutated, and pCDNA3 for *Spalax* p53, were used for normalization (Supplementary Figure S3). In order to investigate the relative contribution of autophagy, 3-MA, an autophagy inhibitor, was used. In parallel, dependence on caspases was assessed using the pan-caspase apoptosis inhibitor Z-VAD-FMK. First, the extent of cell death was determined by the Annexin-PI assays.

**Figure 3:**
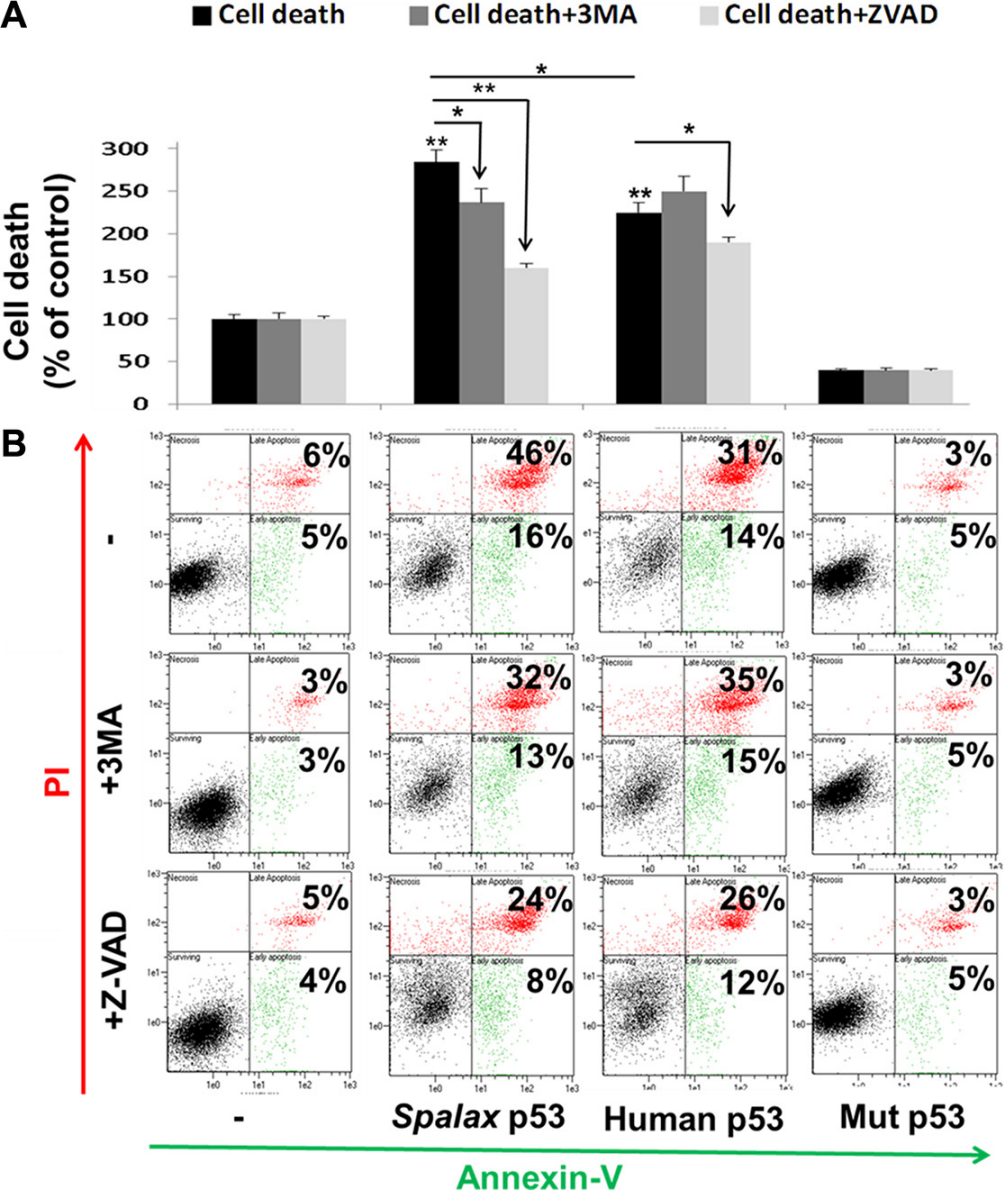
*Spalax* p53 regulates lung cancer cell death via both autophagy and caspases H1299 cells were transfected with wild type human p53, *Spalax* p53 and mutated human H179R p53 (Mut p53) in the presence/absence of an apoptosis inhibitor (Z-VAD-FMK) or authophagy inhibitor (3MA) and examined after 72 hours by Annexin-PI assay (FACS). (**A**) Late apoptosis cell fraction (% of control). (**B**) Representative FACS results. The % of cells in early (An+/PI-, green) and late apoptosis/necrosis (An+/PI+, red) for each treatment are depicted. The experiments were repeated thrice, in duplicates. Results are presented as % of empty vectors ± inhibitors (pCMV for the human wild type and mutated p53; pCDNA3 for the *Spalax* p53). *p < 0.05, **p < 0.005.

Representative Annexin-PI results, presented in Figure 3A, indicate that *Spalax* p53 induced a 2.7-fold increase in cell death. This cell death was prevented by both pathway inhibitors, 3MA and Z-VAD-FMK. This result provides the first indication that cell death caused by *Spalax* p53 in lung cancer cells is not only caspase-dependent but also involves autophagy. In contrast, the 2.3-fold increase in cell death by the human p53 was effectively rescued only in the presence of Z-VAD-FMK and was not influenced at all by treatment with 3MA. The difference between the *Spalax* and human p53′s were significant (*p* < 0.05). Representative Annexin-PI results (Figure 3B) indicate that *Spalax* p53, comparable with the human p53, induced early apoptosis (Annexin+/PI-) and, more profoundly, late apoptosis/necrotic (Annexin+/PI+) cell death.

**Figure 4:**
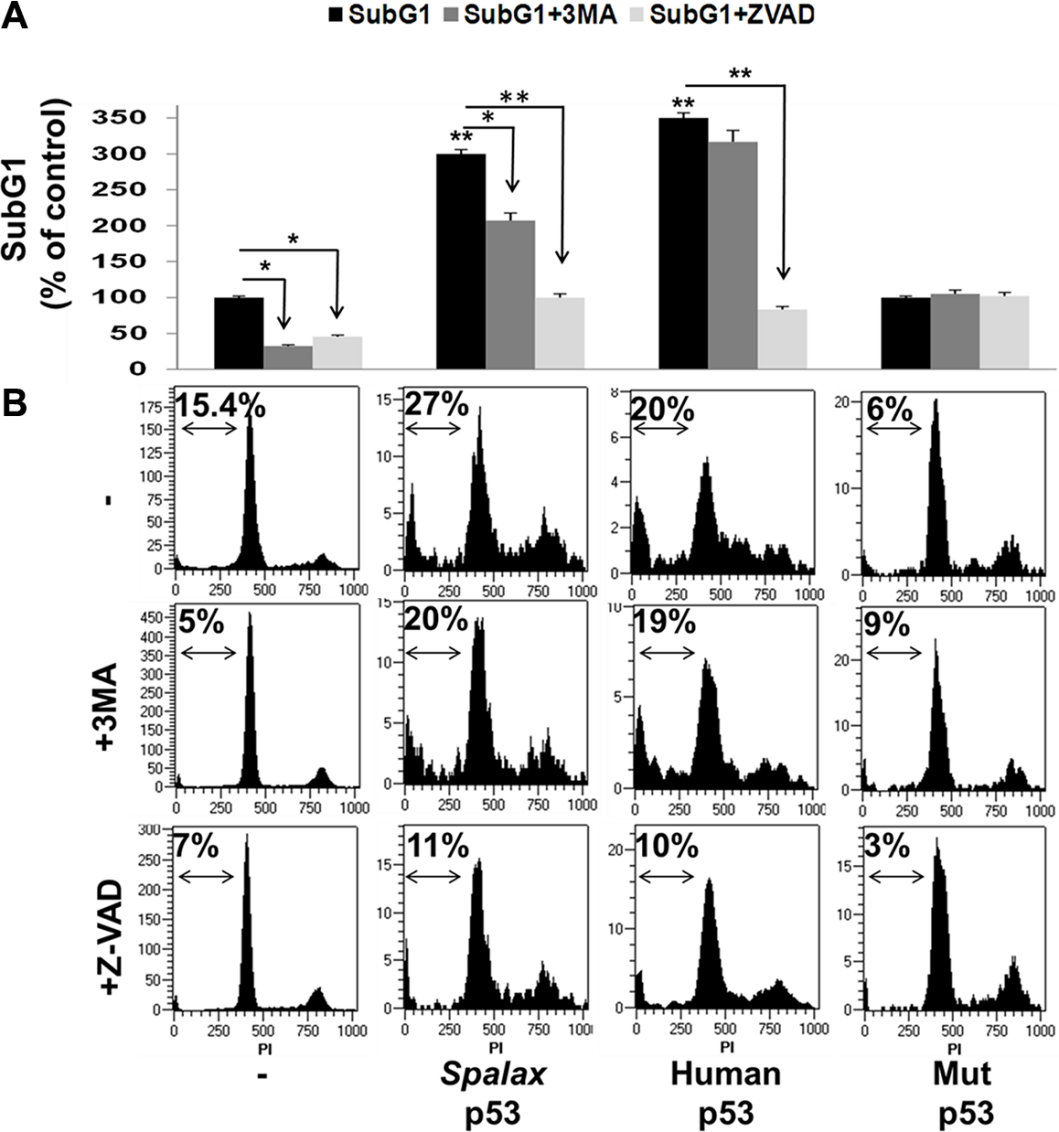
*Spalax* p53 regulates lung cancer SubG1 via both autophagy and caspases H1299 cells were transfected with wild type human p53, *Spalax* p53 and mutated H179R human p53 (Mut p53) in the presence/absence of an apoptosis inhibitor (Z-VAD-FMK) or authophagy inhibitor (3MA) and (**A**) apoptotic cell fraction (SubG1) was examined by cell cycle (FACS) after 72 hours (**B**) Representative FACS results. The % of SubG1 cells for each treatment is depicted. The experiments were repeated thrice, in duplicates. Results are presented as % of empty vectors (pCMV for the human wild type and mutated p53; pCDNA3 for the *Spalax* p53) ± inhibitors. *p < 0,05, **p < 0.005.

The effect of the *Spalax* p53 on cell death was further assessed by measuring SubG1 fraction, indicative of apoptotic cell death, using cell cycle analysis. As detailed above, H1299 cells were transfected with *Spalax* p53 in the presence/absence of Z-VAD-FMK or 3MA. After 72 hours of incubation, cell cycle was analyzed by flow cytometry. Analysis following transfections with wild type human p53 or H179R mutated p53 plasmids served as controls. Appropriate empty vectors, in the presence/absence of both inhibitors, were used for normalization (Supplementary Figure S4). Results from a representative experiment are shown. A basal 15% SubG1 fraction, indicative of apoptotic cell death (Figure 4A), was documented in the non-transfected cells, which was inhibited in the presence of both Z-VAD-FMK and 3MA. The SubG1 cell fraction was significantly induced by the *Spalax* p53 (3-fold). Comparable with the Annexin-PI results, this effect was effectively reversed by both 3MA and Z-VAD-FMK. The human p53 induced a more potent increase in subG1 (3.5-fold) which, similar to the Annexin-PI results, was blocked by Z-VAD-FMK, but not 3MA. No significant difference was observed between the two p53′s and no effect was observed by the mutated p53. Collectively our findings highlight that while the human p53 regulates lung cancer cell death exclusively via caspase-dependence, *Spalax* p53 induction of cell death appears to involve authophagy-dependent mechanism as well as caspases. A representative cell cycle experiment, depicting the subG1 percentage in all treatments, is shown in Figure 4B.

### Inhibition of lung cancer cell proliferation by the *Spalax* p53 is caspase-dependent

**Figure 5:**
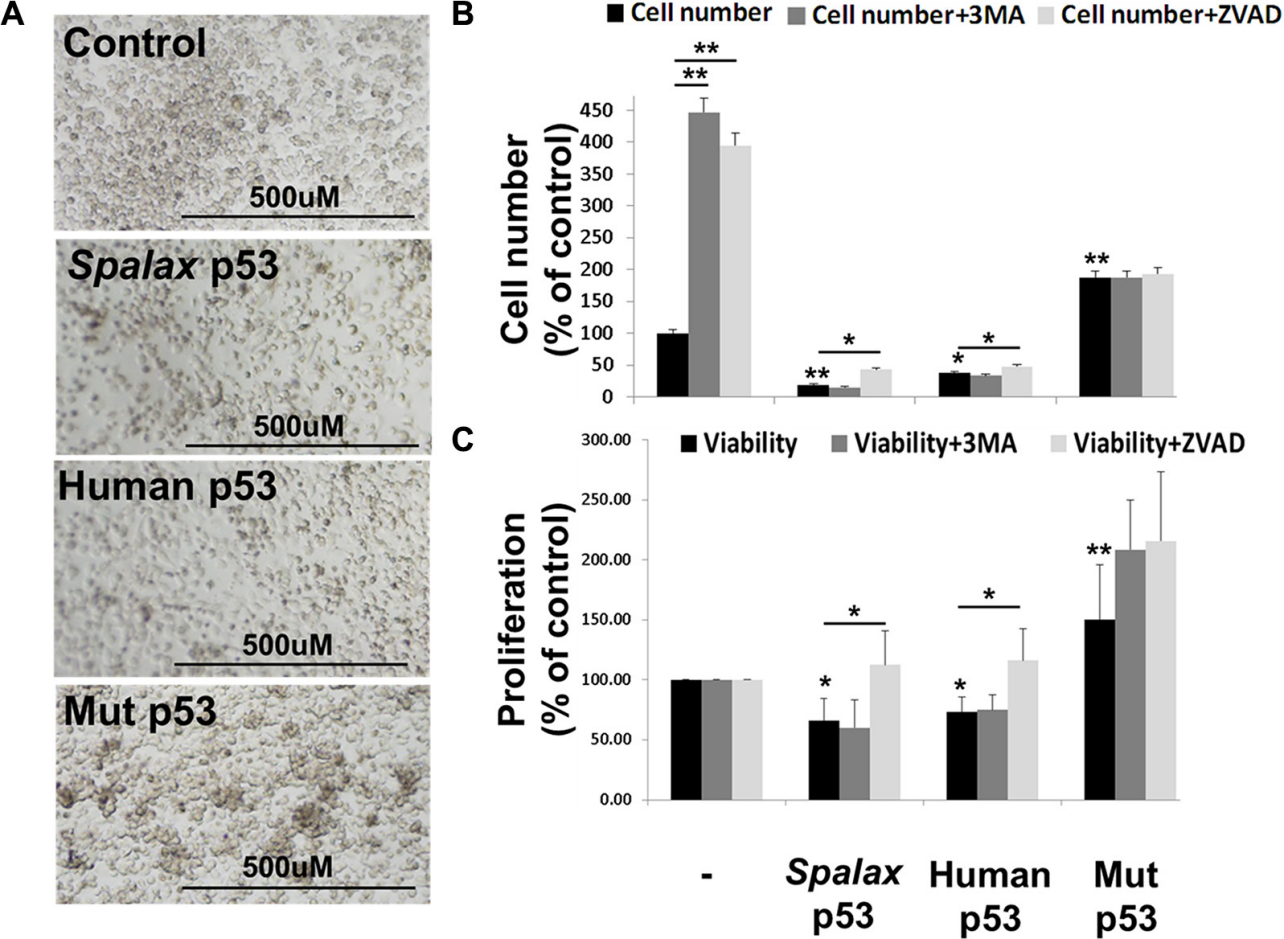
Lung cancer cell proliferation reduction by the *Spalax* p53 is caspase-dependent (**A**) Microscopy images of H1299 cells 72 hours post transfections with *Spalax* p53, wild type human p53 and mutated H179R human p53 (Mut p53). H1299 cells were transfected with the various p53 vectors in the presence/absence of an apoptosis inhibitor (Z-VAD-FMK) or authophagy inhibitor (3MA) and examined for (**B**) Absolute cell number (FACS) and (**C**) WST-1 proliferation assay. Cell counts were repeated twice, WST-1 experiments were repeated three times in duplicates. Results are presented as % of empty vectors ± inhibitors (pCMV for the human wild type and mutated p53; pCDNA3 for the *Spalax* p53). *p < 0,05, **p < 0.005.

Lastly, similar to wild type human p53, lower cell density in the presence of *Spalax* p53 was documented, while higher cell density was shown in the presence of the mutated p53 (Figure 5A). These results inspired us to further examine the role of *Spalax*-p53-induced autophagy on cell proliferation. H1299 cells were transfected with expression plasmids for *Spalax* p53, in the presence or absence of a 3MA or Z-VAD-FMK. Human wild-type p53 or a mutated form of the human p53 (H179R), served as a positive or negative controls, respectively. Appropriate empty vectors, pCMV for human and pCDNA3 for *Spalax* p53, were used in each experiment for normalizations. After 72 hours of incubation, absolute cell counts and cell proliferation were evaluated. In the non-transfected cells, an increase in cell number (Figure 5B) was observed in the presence of both Z-VAD-FMK or 3MA, indicating involvement of both apoptosis and autophagy on cell number in this cancer cell model. In the presence of a plasmid containing a human mutated p53, a 2.8-fold increase in cell number was documented. While wild type human p53 inhibited the number of cells by 43%, *Spalax* p53 had a superior effect, resulting in a potent reduction of 72% in cell number. However, the difference between the two p53′s did not reach statistical significance. The inhibitory effect of *Spalax* p53 on cell number was significantly reversed in the presence of Z-VAD-FMK, but not by 3MA. WST-1 proliferation assay (Figure 5C) further confirmed that *Spalax* p53 reduced cell proliferation by 34% and the human p53 by 28%. This reduction, comparable with the cell counts, was rescued for both p53′s by Z-VAD-FMK, but remained unaffected by the autophagy inhibitor, 3MA. These results suggest that the regulation of cell proliferation in H1299 lung cancer cells by the *Spalax* p53 is exclusively mediated via caspase-dependent mechanisms and does not involve activation of autophagy.

## DISCUSSION

Cells exposed to hypoxic conditions activate wild-type p53 protein, resulting in apoptotic cell death. Hypoxia was suggested as a selection pressure, frequently mutating p53 in tumors, abrogating hypoxia-induced apoptosis. *Spalax* is a blind mole rat that spends its life underground, living, mostly during winter floods, under extreme hypoxic conditions. This superspecies have adapted to severe environment by altering an array of hypoxia-mediated genes [3, 14–18]. By cloning the *Spalax* p53 mRNA a *Spalax*-specific modification was revealed within the p53 DNA binding domain, replacing arginine (R) with lysine (K) at position 172 (corresponding to codon 174 in humans). This specific mutation was reported in tumors of various types, suggesting that the evolution of p53 in hypoxia-stressed *Spalax* mimics human tumor evolution [6]. By using reporter assays, it was further discovered that the *Spalax* p53 protein is transcriptionally biased toward G1 arrest activation and impaired for apoptosis target genes, of both human [6] and *Spalax* [7] origin.

It is now well accepted that apoptosis is not the only form of programmed cell death and that mechanisms that depend on autophagy are also involved [10]. We were therefore interested to investigate the possibility that *Spalax* p53 can activate autophagy. Non small cell lung cancer cells, that are p53-null (H1299), served as the cellular platform. Autophagy is characterized by the formation of acidic vesicular organelles (AVO) in the cell cytoplasm [19], leading to disruption of cytoplasm compartments before nuclear collapse. In our study, we have used the supravital stain acridine orange which accumulates in acidic compartment and is proportional to the degree of authophagy in the cells. A second complementary method to elucidate the level of autophagy in the cells was the GFP-LC3 turnover, an indicator for autophagy flux. We have confirmed, by the two methods, albeit with different potencies in comparison to the human p53, that *Spalax* p53 is able to activate autophagy in the cancer cell model. We have further corroborated the mechanism of *Spalax*-induced autophagy by using bafilomycin A1, an autophagy inhibitor. Activation of autophagy by the human p53, as shown in this current work, was previously reported [20] and suggests to be an integral part of p53 tumor suppressive function [21]. We were not able to conclude, based on the methods used in this work, whether the extent of autophagy initiated by *Spalax* p53 is equal, superior, or inferior, compared with the human wild type p53. It was previously shown that in tissues generated from *Spalax* hearts, under hypoxic conditions, mitophagy, the selective degradation of the mitochondria by autophagy, occurs [22]. Interestingly, in another study, the sub population of *Spalax* galili, living in chalk soil, showed significantly higher protein levels of the autophagy protein ATG7 and of the measure of autophagic flux, based on the LC3-II/LC3-I ratio [23]. Other underground living and burrowing animals, such as the naked mole rat, showed a similar high level of autophagy markers in multiple tissues [24]. Our report is, to the best of our knowledge, the first to stress the direct capability of the *Spalax* p53 to induce macroautophagy and is to report such an action in cancer cells *in vitro*.

In addition to numerous adaptations to underground life, *Spalax* is characterized by a remarkable longevity, with a maximum documented lifespan of 21 years. Further studies have established that the blind mole rat is highly cancer resistant [12]. Among thousands of captive animals over a 40-year period, not even a single case of spontaneous tumor have been developed, including in over 20 years old animals that may be more vulnerable to cancer. Moreover, chemical carcinogens failed to induce tumor growth in *Spalax*, while tumors were clearly evident in other rodents and primary *Spalax* fibroblast inhibited growth and killed cancer cells, but not normal cells, either through direct fibroblast-cancer cell interaction or via soluble factors [12]. Yet, the effect of *Spalax* p53 on the fate of cancer cells is a research direction that was largely overlooked. This is since early studies of the *Spalax* p53 primarily aimed to evaluate the transcription factor activities of this specific p53 protein [6]. Consecutive studies have further explored the transcription of additional target genes by the *Spalax* p53 under normoxic versus hypoxic environment [7, 25–27]. We were therefore interested to study the direct anticancer effect of the *Spalax* p53. Such anti-cancer effects by wild type human p53 [28–30] and the mitogenic effects by codon 179 mutated p53 [28, 31] in H1299 cells correspond with our current results. We have further confirmed that *Spalax* p53 can significantly inhibit lung cancer cell proliferation and induce potent cell death *in vitro*. Support that the *Spalax* p53, that was shown to be transcriptionally biased against apoptotic target genes, is able to induce cell death *in vitro*, was previously demonstrated by our group in a murine pro B (Ba/F3) cell line [7]. In this leukemia cell model, *Spalax* p53 was less potent in inducing SubG1, compared to human p53. Similarly, in this current work, in the non-small cell lung cancer cells, a solid tumor model, induction of SubG1 by the *Spalax*-p53 was somehow lower compared with the effect by the human p53. However when we analyzed the extent of cell death by another methods, Annexin-PI, the impact of *spalax* p53 on cell death was higher compared to human wild type p53. The discrepancy between the two methods (cell cycle and Annexin-PI) may be explained by the observations that phosphatidylserines externalization and binding of Annexin-V may occur not only in apoptosis, but also in other types of cell death including necrosis [32] and autophagy [33–35]. Therefore while SubG1 analysis by cell cycle is indicative only for apoptotic cell death, positive signal with Annexin V staining can be considered a marker of any type of cell death and may provide additional valuable information [36]. In another recent study, *S. galili* p53, sibling species with the *S. judaei*, failed to induce early apoptotis (Annexin+/PI-) in H1299. Nonetheless, in this experiment the cells were analyzed 24 hours post transfections, and not 72 hours as in our study and no data regarding late apoptosis (Annexin+/PI+), which was the main cell death observed in our system, was presented. In addition, although the *S. galili* and the *S. judaei* p53 share the same protein sequence, carrying an R172K mutation, there are two synonymous variations between the two species [9]. The ability of p53 of other mammals of the Tibet plateau, the wild zokor (*Myospalax baileyi*) and root vole (*Microtus oeconomus*), to induce apoptosis in cervical cancer cells, was demonstrated as well [37].

Lastly, based on our observation that the *Spalax* p53 can activate autophagy, we were particularly interested to assess the relative contribution of autophagy versus caspases on cancer cell proliferation and death. By using an autophagy inhibitor (3MA) or a pan caspase inhibitor (Z-VAD-FMK), it emerged that inhibition of cell proliferation by the *Spalax* p53, similar to the human p53, is solely caspase-dependent. In contrast, while the induction of cell death by the human p53 was utterly caspase-mediated, the death induced by the *Spalax* p53 had a mixed phenotype of autophagy and caspase-dependence. These results propose that there is a distinction between the two p53′s with regards to the mechanisms through which cancer cell death is promoted.

The observation that the inhibition of autophagy rescued cancer cells from death induced by the *Spalax* p53 provided the first evidence to substantiate that by activation of the autophagic pathway, *Spalax* p53 may directly eliminate cancer cells. It is well established that elimination of cancer cells may occur not only via apoptosis but could also be mediated by autophagy-dependent mechanisms. Support for that came from studies in which a decline in autophagic activity was related to tumorigenesis, whereas autophagy activation led to cancer cell death [10, 19, 20, 38–40]. We have therefore hypothesized that, as part of the cancer resistance phenotype, *Spalax* p53 may be efficient in inducing cancer cell death that is autophagy, as well as caspase-dependent. It is now clear that caspases are a molecular switch node in the crosstalk between autophagy and apoptosis [41, 42].

Similarly, human p53-induced-autophagy was recently shown to contribute to the execution of p53 dependent apoptosis, indicating that p53 can promote cell death through multiple pathways [13]. Indeed, autophagy and apoptosis often occur in the same cell, mostly in a sequence in which autophagy precedes apoptosis [43–45]. These observations are in accord with a recent report indicating that autophagy can lead to the execution of apoptotic (type I) or necrotic (type III) cell death [39], by depleting endogenous inhibitors of these cell death pathways [43]. This further suggests that, even though apoptosis is part of the mechanisms through which the cells are finally destroyed, autophagy can also mediate cell elimination as a genuine effector mechanism [40]. As p53 was shown to transcriptionally activate several target genes that result in the activation of autophagy [43, 46], assessments of a similar action by the *Spalax* p53 is merited.

To conclude the blind more rat, *Spalax*, shows a striking resistance to cancer. Our recent findings suggest that this resistance phenotype may be partly mediated by *Spalax*‘s unique p53 protein, which seems to retain potent and efficient cancer death activities, which are mainly autophagy mediated and caspase-dependent.

## MATERIALS AND METHODS

### Plasmids

For transfection studies, we have used expression plasmids of the human p53, human mutated p53 (H179R), and *Spalax* p53, as previously described [6, 8]. The human wild-type p53 was ligated into a pCMV (cytomegalovirus) construct. *Spalax* p53 cDNA was cloned from mRNA prepared from a whole embryo belonging to the S. judaei superspecies and was ligated into a pCDNA3 expression vector. Empty vectors were used appropriately for normalization in all experiments.

### Reagents and chemicals

Pan-caspase specific apoptosis inhibitor (Z-VAD-FMK, ALX-260-020-M001, Enzo Life Sciences). Authophagy inhibitor (3MA), Bafilomycin A1 (B1793) and Acridine Orange (A6014) from Sigma Aldrich. 30% Hydrogen peroxide (H_2_O_2_) was from Merck Millipore (107209).

### Cell culture

H1299 cells (human non-small cell lung cancer, ATCC HTB-96) were grown in 10% FCS in RPMI medium 1640 (Sigma) supplemented with 2 mM glutamine, 100 μg/ml streptomycin, and 100 units/ml penicillin at 37°C in a humidified incubator with 5% CO_2_. The cells were tested negative for mycoplasma contamination using the e-Myco PCR mycoplasma detection kit (25235, iNtRON Biothechnology).

### Transient transfections

H1299 cell lines were plated (2 × 10^4^/96 well plates). Transient transfection was performed the next day using JetPEI transfection reagent (101–10 N) according to manufacturer's instructions (Polyplus Transfection). Cells were transfected with 1 μg of the various *p53* plasmids in the presence/absence of pan-caspase specific apoptosis inhibitor (50 μM Z-VAD-FMK) or authophagy inhibitor (10 mM, 3MA). Appropriate controls were performed for each assay and included vehicle only and transfections with empty vectors (pCDNA3 or pCMV) in the presence/absence of the apoptosis or authophagy inhibitors. Cell were analyzed by the methods detailed below, 72 hours post transfections.

### Flow cytometry

MACSQuant (Miltenyi Biotec) was used. For absolute cell number, the cells were harvested 72 hours post trasnfections, in a fixed volume and counted. For annexin-PI assay, cells were harvested 72 hours post trasnfections and incubated with Annexin v-FITC and PI (K101–400, BioVision Inc.) according to manufacturer's instructions. Annexin−/PI−, surviving cell fraction; annexin+/PI−, early apoptosis; and annexin+/PI+, late apoptosis/necrosis. For cell cycle analysis the cells were harvested, 72 hours post trasnfections, permeabilized by 70% ethanol for 20 min at −20°C and stained for 15 min at room temperature with DNA propidium iodide (PI) (50 μg/mL)/RNAse A (10 μ g/mL) (P4170, Sigma-Aldrich).

### Proliferation assay

WST-1 (Roche, Indianapolis, IN, USA; 10% final concentration) was incubated with cells at 37°C for 1 h and read with a microELISA reader at 440 nm.

### Supravital cell-staining with acridine orange for autophagy detection

Acridine orange was added at a final concentration of 1 μg/ml for a period of 15 minutes to the cells, 72 hours post trasnfections. Bafilomycin A1, an autophagy inhibitor, dissolved in DMSO, was added to the cells 30 min before addition of acridine orange. Appropriate negative (empty vectors) or positive controls (cells treated with 3% H_2_O_2_) were done. The cells were then washed twice with PBS. Pictures were obtained with a fluorescence microscope. Fluorescence was quantified using NIH ImageJ software.

### Quantification of GFP-LC3 levels

The turnover of GFP-LC3 (reflecting an autophagic flux) is a reliable and simple assay to measure autophagic activity in living mammalian cells [47]. Enhanced autophagic flux is detected as a decreased total cellular GFP signal. GFP fused to the N terminus of LC3 (a generous gift from Prof. Zvulun Elazar, The Department of Biological Chemistry, The Weizmann Institute of Science, Rehovot, Israel). The GFP-LC3 plasmid was transfected in the presence/absence of the various p53 plasmids, as described above. The levels of GFP in the cells was quantified 72 hours post transfection, by flow-cytometry (MACSQuant).

### Light and fluorescent microscopy

The cells were visualized, 72 hours post trasnfections, by a light and fluorescence microscope equipped with a camera (model IX71; Olympus) with a × 20/0.50 objective lens and Cell^A (version 3.1) Olympus software imaging. Fluorescence was quantified using NIH ImageJ software.

### Statistical analysis

Experiments were analyzed by a two-sided Student's unpaired *t*-test for significance (*P* < 0.05).

## SUPPLEMENTARY MATERIALS FIGURES


