# Supplementary material for: The double benefit of Spalax p53: surviving underground hypoxia while defying lung cancer cells in vitro via autophagy and caspase-dependent cell death
Source: Oncotarget. 2016 Aug 20;7(39):63242–51. doi: 10.18632/oncotarget.11443 (PMC5325360; doi:10.18632/oncotarget.11443)
Supplement: Supplementary file 1 [file oncotarget-07-63242-s001.pdf]

The double benefit of *Spalax* p53: surviving underground hypoxia while defying lung cancer *cells in vitro* via autophagy and caspase-dependent cell death

Supplementary Materials

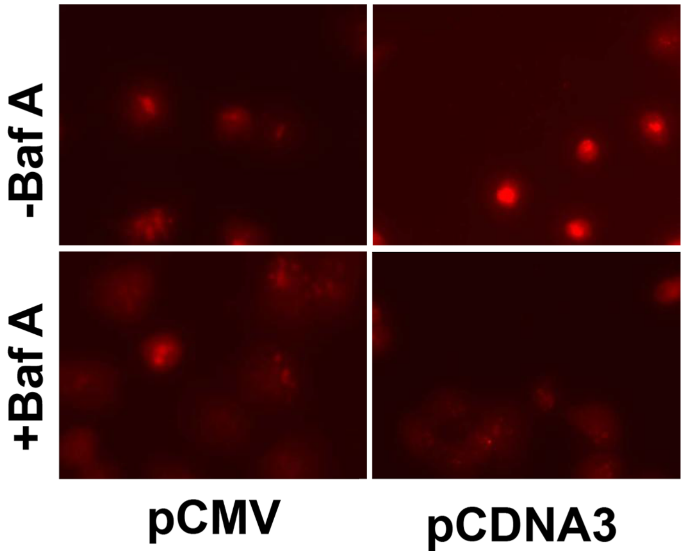

Supplementary Figure S1: Representative fluorescent microscopy images of H1299 cells transfected with empty pCMV or pCDNA3 plasmids. After 72 hours the cells were stained with acridine orange in the presence or absence of an autophagy inhibitor, Bafilomycin A1 (Baf A).

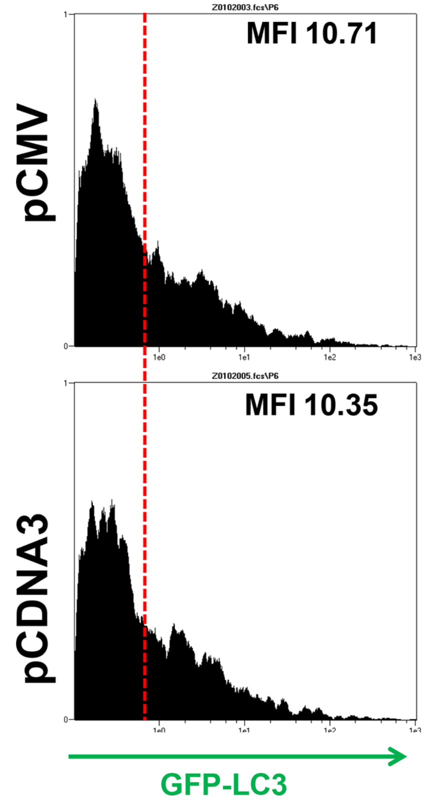

Supplementary Figure S2: Representative FACS histogram with MFI values for cells co-transfected with empty vectors (pCMV for the human p53; pCDNA3 for the *Spalax* p53) and GFP-LC3.

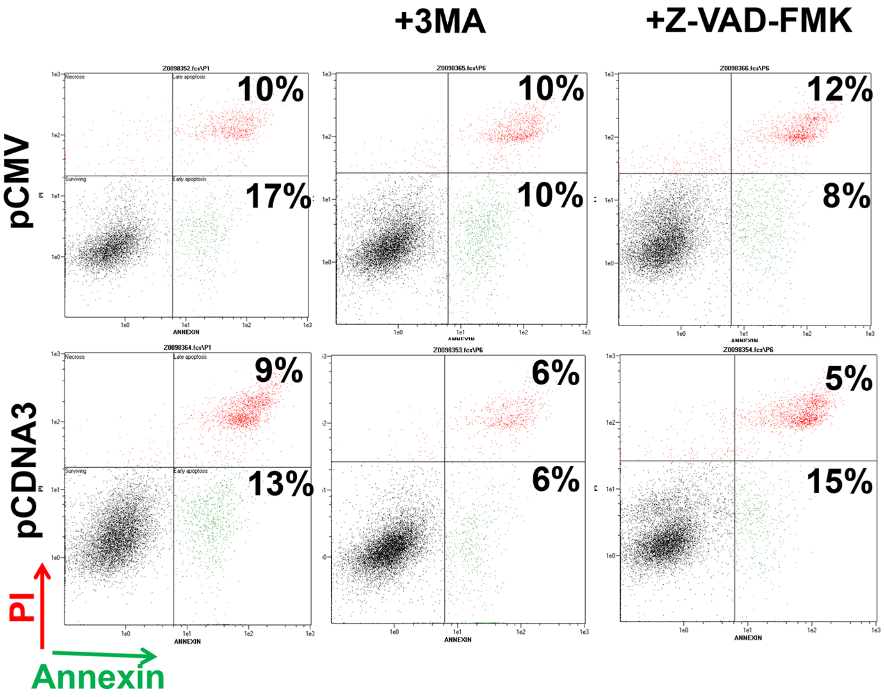

Supplementary Figure S3: Representative Annexin-PI results of H1299 cells transfected with pCMV or pCDNA3 in the presence/absence of an apoptosis inhibitor (Z-VAD-FMK) or authophagy inhibitor (3MA) after 72 hours. The % of cells in early (An+/PI−, green) and late apoptosis/necrosis (An+/PI+, red) for each vector are depicted.

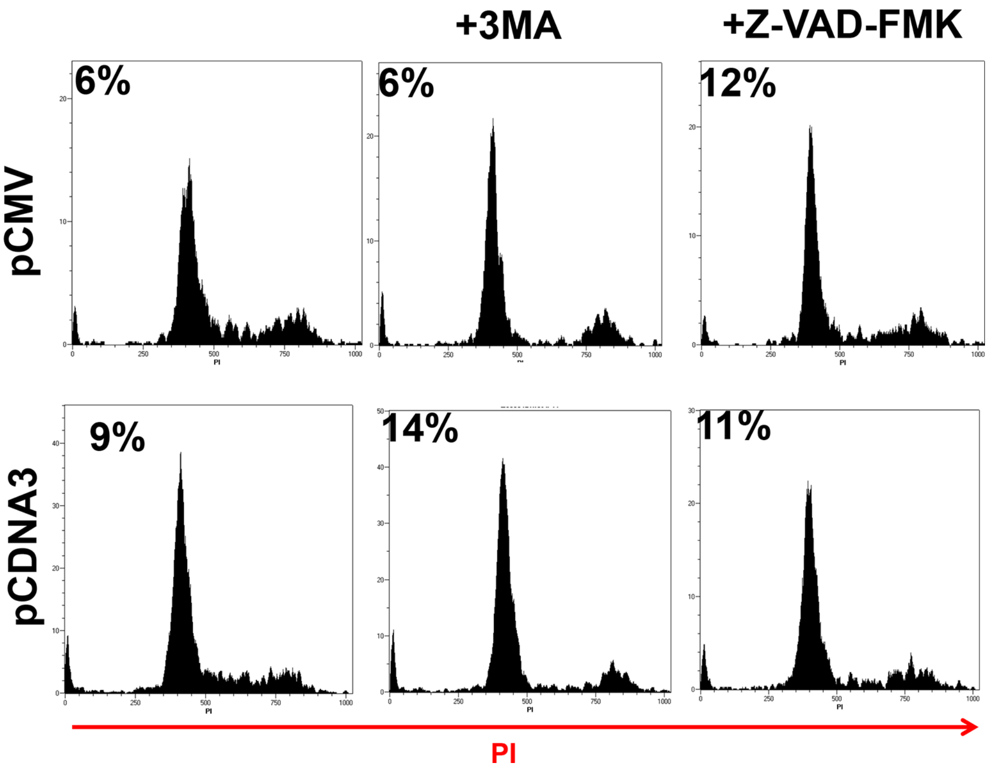

Supplementary Figure S4: Representative cell cycle analysis of H1299 cells transfected with pCMV or pCDNA3 plasmids in the presence/absence of an apoptosis inhibitor (Z-VAD-FMK) or authophagy inhibitor (3MA). The % of SubG1 cells for each treatment is depicted.
